# Few long-term consequences after prolonged maternal separation in female Wistar rats

**DOI:** 10.1371/journal.pone.0190042

**Published:** 2017-12-21

**Authors:** Stina Lundberg, Klas S. P. Abelson, Ingrid Nylander, Erika Roman

**Affiliations:** 1 Department of Pharmaceutical Biosciences, Uppsala University, Uppsala, Sweden; 2 Department of Experimental Medicine, University of Copenhagen, Copenhagen, Denmark; Oregon Health and Science University, UNITED STATES

## Abstract

Environmental factors during the early-life period are known to have long-term consequences for the adult phenotype. An intimate interplay between genes and environment shape the individual and may affect vulnerability for psychopathology in a sex-dependent manner. A rodent maternal separation model was here used to study the long-term effects of different early-life rearing conditions on adult behavior, HPA axis activity and long-term voluntary alcohol intake in female rats. Litters were subjected to 15 min (MS15) or 360 min (MS360) of daily maternal separation during postnatal day 1–21. In adulthood, the behavioral profiles were investigated using the multivariate concentric square field™ (MCSF) test or examined for HPA axis reactivity by cat-odor exposure with subsequent characterization of voluntary alcohol intake and associated changes in HPA axis activity. Adult female MS360 offspring showed mostly no, or only minor, effects on behavior, HPA axis reactivity and long-term alcohol intake relative to MS15. Instead, more pronounced effects were found dependent on changes in the natural hormonal cycle or by the choice of animal supplier. However, changes were revealed in corticosterone load after long-term alcohol access, as females subjected to MS360 had higher concentrations of fecal corticosterone. The present findings are in line with and expand on previous studies on the long-term effects of maternal separation in female rats with regard to behavior, HPA axis activity and voluntary alcohol intake. It can also be a window into further studies detailing how early-life experiences interact with other risk and protective factors to impact the adult phenotype and how possible sex differences play a role.

## Introduction

Environmental factors, in addition to individual genetics, influence health and vulnerability for disease. For instance, studies in humans have shown that adverse experiences during childhood, such as parental loss, neglect or abuse, can affect neurobiology and behavior [1,2]. Furthermore, evidence from preclinical, epidemiological and clinical studies has demonstrated that adverse early-life experiences can disrupt developmental processes and thereby increase the vulnerability for later psychopathology [3–5]. Reports on early-life experiences have also highlighted protective factors that can promote resilience to adversities and diminish vulnerability [6–8]. Various advancements in studies investigating gene-environment interactions have improved the understanding of risk, protection and resilience in humans and experimental animals [8–10]. Interestingly, sex differences have been described in prevalence and severity of a broad range of conditions and disorders [11,12].

During the postnatal period, the infant is dependent on the mother or primary caregiver, not only for nursing and protection but also for normal development of physiology and behavior [13–15]. Exploring environmental influences during this period can help to gain a greater understanding of early-life influences on developmental trajectories and subsequent adult behavior and physiology. Rodent maternal separation (MS) models are frequently used in order to investigate the impact of early environmental factors on adult neurobiology and behavior [10,16–18]. A short period of daily MS (usually 3–20 min), mimics the behavior of wild rats, where the dam has to leave her litter for foraging, and is used to simulate a safe early environment. Prolonged periods of MS (usually >180 min), rarely occurs in the wild and is used to simulate an adverse early environment. Additional groups can be added to the paradigm to examine the specific effects of handling and/or separation. Several protocols are currently in use and the results are sometimes inconsistent, especially after prolonged periods of MS [16,18,19]. In addition, the mechanism behind the effects of MS remains unclear, but most theories involve changes of the HPA axis and its responsiveness to stress [14,17,18,20].

We have established a MS protocol including short (15 min; MS15) and prolonged (360 min; MS360) periods of daily MS for studies of the long-term effects of early environment on brain function, voluntary alcohol intake and behavior in rats. Using this model, we have provided evidence for long-lasting neurochemical alterations and effects on voluntary alcohol intake and behavior in adult male rats [10,17,21]. The effects in MS360 males result in an adult behavioral phenotype that resembles that of certain lines of alcohol-preferring rats [22,23] and the subset of alcohol use disorder patients characterized by high novelty seeking and impulsivity [24]. The male MS360 rats display higher exploratory activity and altered risk-assessment and risk-taking behavior in adulthood compared to standard reared rats [25], in addition to changes in the endogenous opioid system similar to that of alcohol-preferring rats [17,26–28]. Lastly, adult MS360 males have increased voluntary alcohol intake and a preference for higher alcohol concentrations compared to MS15 males [10,29]. Interestingly, a number of long-term effects detected in male rats appear to be absent or less pronounced in female rats. For instance, prolonged periods of MS have less or no effect on voluntary alcohol intake, neurochemistry and behavior compared to adult standard reared [30–33] and MS15 [31–33] female rats. In addition, there are fewer studies examining the impact of MS in female rats, possibly due to a combination of sex- [34,35] and publication biases [36,37].

The aim of the present study was to further explore the consequences of prolonged MS on adult behavior, alcohol intake as well as basal and challenged HPA axis activity in female rats. In Experiment 1, the adult behavioral profile was assessed using the multivariate concentric square field™ (MCSF) test, together with determination of the estrus cycle stage at time of testing. The guiding principle for the MCSF test is that it is unprejudiced, i.e. the test is not designed to measure a particular mental condition. The MCSF test investigates the animal’s exploratory strategy when exposed to a diverse, novel environment which includes opportunities for exploration, risk assessment, risk taking and shelter seeking, and thereby provides a behavioral profile [38,39]. Experiment 2 was focused on alcohol intake and HPA axis activity, using animals from two different suppliers. Basal and challenged HPA axis activity was assessed in adult females before and after long-term voluntary alcohol intake in an attempt to reveal any latent differences in HPA axis function. Animals from different supplier origin were used to further try to tease apart the possible resilience effect. Supplier origin has in standard reared, adult males been shown to affect behavior, neurobiology and voluntary alcohol intake [40–44], and a recent study discovered a nonsense mutation resulting in loss of mGlu2 receptor function, which appears in different frequencies in rats from different suppliers [45]. Whether females exhibit supplier-dependent differences and how they affect the vulnerability to early-life experiences is not known.

## Materials and methods

**Fig 1:**
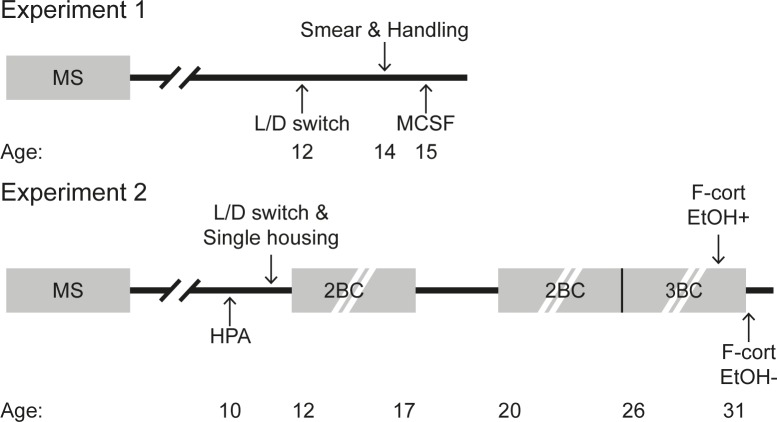
Overview of the experimental procedures. Adult behavior was assessed in Experiment 1 and adult HPA axis activity and long-term voluntary alcohol intake were assessed in Experiment 2. The animals’ age in weeks is given for the different procedures. *Abbreviations*: MS, maternal separation; L/D switch, light/dark cycle reversal; MCSF, multivariate concentric square field™ test; HPA, HPA axis reactivity test; 2BC, alcohol access in the two-bottle free-choice paradigm; 3BC, alcohol access in the three-bottle free-choice paradigm; F-cort EtOH+, collection of fresh fecal boli directly after the three consecutive days of alcohol access; F-cort EtOH-, collection of fresh fecal boli after four days of water only.

An overview of the experimental procedures in Experiments 1 and 2 are summarized in Fig 1.

### Animals and housing

Pregnant Wistar rats (see Table 1 for supplier information) arrived at the animal facility on gestational days 12–16. Upon arrival, the dams were single-housed in standard transparent cages type IV (59 × 38 x 20 cm) with raised lids, containing wood chip bedding and paper sheets (Cellstoff; Papyrus, Möndal, Sweden) as nesting material. Throughout all experiments, the animals were maintained on standard pellet food (type R36; Lantmännen, Kimstad, Sweden) and tap water *ad libitum*. All animals were housed in temperature- (22 ± 1°C) and humidity-controlled (50 ± 10%) animal rooms on a normal 12 h light/dark cycle with lights on at 06:00 (Exp. 1) or 07:00 (Exp. 2). All animal experiments were performed according to protocols approved by the Uppsala Animal Ethics Committee (ethical approvals C33/2, C427/12 and C14/14) and in accordance with the Swedish Animal Protection Legislation (SFS 1998:56) and the European Communities Council Directive (Exp. 1: 86/609/EEC) or the European Union Directive (Exp.2: 2010/63/EU).

**Table 1:** Overview of the Wistar rat suppliers used, including nomenclature and abbreviations used in this paper.

Experiment	Supplier	Location	Nomenclature	Abbreviation in this paper	*n*, offspring (MS15/MS360)
1	Scanbur BK	Sollentuna, Sweden	Sca:WI	Sca	15/15
2	Envigo (former Harlan Labs)	Horst, the Netherlands	RccHan:WI	Rcc	10/10
	Taconic Biosciences	Ejby, Denmark	HanTac:WI	Tac	10/10

### Maternal separation

Animals were reared in one of two rearing conditions: daily separation for 360 min, used as experimental condition to simulate adverse environment, or the control condition, where litters were handled equally but only separated for a short period of time (15 min). Short separation was used as control to ensure similar handling of litters and that any detected differences would be due to difference in duration of separation only.

On the day of birth (postnatal day, PND, 0), the sex of the pups was noted and they were then cross-fostered into litters of 9–10 pups (males, n = 5–6; females, n = 3–5). The litters were then randomly assigned to one of two rearing conditions: daily 15 min (MS15, Exp. 1: *n* = 9 litters; Exp. 2: *n* = 4 litters) or 360 min (MS360, Exp. 1: *n* = 10 litters; Exp. 2: *n* = 4 litters) of MS.

The separations occurred once daily during PNDs 1–21. First the dam and then the pups were removed from the home cage. Each litter was placed together in transparent cages type II (22 × 16 × 14 cm) containing wood chip bedding and moved to an adjacent room with similar conditions as the animal room but with higher temperature (Exp. 1: 24 ± 0.2°C; Exp. 2: 30 ± 0.5°C). During the separations, the dams in the MS15 group were moved to other cages and the litters were returned before the dams. In the MS360 group, the dams were returned to the home cages during the separations, and removed again before the litters were returned. Separation sessions were performed during the light period, with the first MS15 litter started at 09:00 and the first MS360 litter started at 09:30. Home cages were changed twice during PNDs 1–21 for both groups, with a small amount of old bedding material mixed in with the clean bedding. The litters were weighed every third to fourth day, on PNDs 1, 4, 7, 10, 13/14, 16/17 and 19/20. All litters were also weighed on the day of weaning on PND 22 (the litter weight data can be found in references [29] (Exp. 1) and [46] (Exp. 2)). Thereafter, the animals were housed in same-sex and -experimental groups with 3–5 rats per cage in standard transparent cages type IV (59 × 38 × 20 cm) with raised lids. Cages were changed once a week.

### Experiment 1 –behavior

At 12 weeks of age, 1–2 females per litter (Sca) were randomly selected and used for assessment of behavioral profiles in the MCSF test.

#### Determination of estrous cycle phases and general test procedure

The females (*n* = 15 rats/group) were transferred to an animal room with a reversed 12-hour light/dark cycle (lights off at 09:00). The animals were allowed to adapt to the reversed light/dark cycle for two weeks [47]. Starting at 14 weeks of age, daily vaginal smears were collected using the lavage technique in order to determine stable estrus cycling [48]. Smears were collected at 13:00 and immediately analyzed under a microscope (magnification ×100) and scored according to the predominant cell type. Characterization of the estrus cycle according to predominate cell type in the vaginal smears enables reliable identification of the stages, i.e. proestrus (nucleated epithelial cells), estrus (cornified epithelial cells), metestrus (leucocytes in addition to nucleated cells and shrinking cornified cells) and diestrus (leucocytes) [48].

In addition to the individual handling when vaginal smears were taken, the animals were adapted to a transportation bucket used for transportation of the animals between the animal room and the test arena. At 15 weeks of age, the behavioral profile of the animals was investigated using the MCSF test. The rats were tested in a pseudorandom order with each experimental group being distributed evenly over the different test days and times to avoid time and order bias. All testing was performed in an adjacent room with similar conditions as the animal room. Observations were carried out during the dark period of the light/dark cycle, starting at 13:00. On the day of behavioral testing, vaginal smears were collected immediately after the MCSF test in order to determine the estrus cycle stage during the test session, at the same time the body weights of the animals were noted.

#### The MCSF test

The MCSF test has been described in detail elsewhere [39]. The MCSF provides several areas for the animal to explore (see S1 Fig) including sheltered, open and elevated areas, a hole-board device and areas with different illumination. The apparatus is 70 × 70 cm with a walled off center (41 × 41 cm) from which three corridors can be accessed through openings in the walls. In one corner, accessible from two of the corridors, is the hurdle, an elevated platform (10 cm above the floor) with a hole-board device with two holes (2.5 cm in diameter) and a photocell underneath, which records the number of nose pokes into the holes. In the opposite corner is the dark corner room (DCR), a smaller, covered area, to which the animal has access through one entrance. Along the fourth side of the arena is the bridge, a stainless-steel wire-mesh construction (10 mm between bars) that spans over an illuminated opening in the floor and is accessible from one of the corridors via the slope. In addition, during analysis a central circle (CTRCI) is added in the center. Lighting conditions (lux) in the arena were as follows: center and corridors <30, DCR <0.5 and bridge >500.

The animal to be tested was transferred in a bucket from the home cage to the MCSF apparatus and released in the center facing the wall without an opening. The test session lasted for 20 min. After each test, the floor of the arena was wiped with a cloth containing 10% alcohol. Sufficient time was allowed for the floor to dry before the next animal was placed in the arena.

#### Behavioral recordings

The animals were recorded from above and monitored from an adjacent room. Rearing, grooming and stretched attend postures (SAPs, between corridor-hurdle, corridor-DCR and slope-bridge) were scored by direct observation and the number of fecal boli and urinations were noted after each animal. Manual scoring of the behavior in the MCSF test was performed using the software Score (Pär Nyström, Copyright Soldis, Uppsala, Sweden) by an observer blinded to the experimental group. A visit to a zone was only scored as such if both hind legs had crossed into that section. The latency (L, seconds) of first visit to a zone, frequency (F) of visits and duration (D, seconds) of time spent in a certain zone were registered. The EthoVision system (version 2.3, Noldus Information Technology, Wageningen, The Netherlands) was used to measure velocity (cm/s) and distance (cm) in the MCSF arena. In addition, the following parameters were derived: the latency to leave the center (L leave); the sum of all zone visits (TOTACT); the sum of all SAPs (SAP total); the frequency and duration spent in the corridors (F and D TOTCORR); the duration per visit (D/F) and percental frequency and duration (%F and %D) for all zones; the F and D risk/shelter indices ((F bridge-F DCR)/(F bridge+ F DCR) and (D bridge-D DCR)/(D bridge+ D DCR)); and the slope/bridge interval ((L slope-L bridge)/L slope).

### Experiment 2 –HPA axis activity and long-term voluntary alcohol intake

The adult females included in Experiment 2 (*n* = 10 rats/group; Rcc and Tac, whole litters), were also tested as adolescents together with their male siblings; the procedures and results can be found in [46].

#### HPA axis reactivity

At 10 weeks of age, baseline and challenged levels of corticosterone were assessed. As challenge, cat-odor exposure during 30 minutes of isolation was used; each animal was isolated in a cage (type III: 42.5 × 26.5 × 18 cm) containing bedding and an odor tube with cat litter and fur-saturated fleece. The odor tubes were prepared with fresh cat litter and newly used fleece blankets, each tube was topped with lightly packed cotton, sealed and frozen at -18°C. Shortly before use, the tubes were thawed and opened when placed into the isolation cages, each tube was only used once. Odor-exposure and blood sampling were performed in separate rooms adjacent to the animal room, under the same environmental conditions. An experimenter was not allowed to enter the animal room after entering the odor-exposure room on the same day and several doors separated the animal room from the odor-exposure room.

Blood samples for corticosterone analysis were collected immediately before and after the odor-exposure, and after 120 minutes of recovery in the home cage. All samples were collected during the light phase with baseline samples collected between 09:00–10:40. Blood samples were collected by needle puncture of hind paw veins (Microvette 100 μl, Sarstedt AG & Co). After the last blood sample, the animals were weighed and the body weights were noted. Serum extraction and radioimmunoassay analysis were performed as previously described [46].

The week after the HPA axis reactivity test, the animals were single-housed in transparent cages type III (42.5 × 26.5 × 18 cm), containing bedding and paper sheets, and were moved to a room with reversed 12 h light/dark cycle with lights off at 07:00, and left to acclimatize for one week.

#### Long-term voluntary alcohol intake

At 12 weeks of age, the animals received access to alcohol in a two-bottle free-choice (20% v/v alcohol and water) paradigm in a modified intermittent schedule with alcohol access three consecutive days per week followed by four days of water only [49]. The alcohol access started during the dark phase, and the alcohol and water was changed before access each day. The placement of the bottles was rotated to avoid any side bias.

The alcohol access followed the modified intermittent schedule for six weeks, followed by an alcohol deprivation period of two weeks. Alcohol access was then reinstated, and continued as described above for an additional six weeks.

After the weeks on two-bottle free-choice a third bottle of 5% alcohol (v/v) was added to the paradigm to investigate the alcohol preference dynamics between the two alcohol concentrations, and if rearing condition or supplier affect the choice of preferred concentration. At the same time, the animals were transferred to larger cages (type IV; 59 × 38 × 20 cm) to accommodate the third bottle and due to the fact that the body weights of the animals were approaching the weight *versus* housing area limit for the type III cage size. The animals continued on the three-bottle free-choice paradigm on the modified intermittent schedule for six weeks. The experiment was then terminated and the animals euthanized the week after last alcohol access at 32 weeks of age.

Alcohol and water intake was measured for every access day and the animals were weighed once a week. The alcohol intake (g/kg), alcohol preference (%) as well as water and total fluid intake (g/kg) were calculated for each access day and summarized in weekly averages. For the three-bottle free-choice period, both concentration-dependent and total alcohol intake and preference were calculated. The two- and three-bottle free-choice periods were analyzed separately for the alcohol intake and preference, while water- and total fluid intakes were analyzed for the whole alcohol access period together.

#### Fecal corticosterone

Fecal corticosterone is a measure of the cumulative corticosterone load over the time pending the excretion of the fecal boli, with an approximate lag time from release in blood to excretion into feces of 8–24 h [50–54]. Fresh fecal boli were collected at two time points: at removal of the alcohol bottles on the third day of access in week 19, as a measure of the corticosterone load during alcohol access; and after the four days of water only in week 20, as a measure of the corticosterone load without alcohol access. The freshest boli (0.8–2.4 g) were collected and quickly frozen at -18°C until analysis. The samples were processed and analyzed as described previously [55–57].

### Statistical analysis

Statistical operations were carried out in R 3.2.3 [58] using the nparLD package [59] for analysis of main effects and interactions in non-parametrical, longitudinal data sets and in Statistica 13.2 (Dell Inc., Tulsa, OK, USA) for all other analyses. Parameters were examined for normality using the Shapiro-Wilk’s W test. The body weight and HPA axis reactivity data sets were found to have normal distributions and thus, parametric statistics were used. All other data sets: MCSF parameters, alcohol access data and fecal corticosterone, did not show normal distributions and consequently, non-parametric statistics were used.

#### Parametric statistics

Body weights at the time of MCSF testing in Experiment 1 were evaluated with Student’s unpaired t-test. For the longitudinal body weight measurements in Experiment 2 and the HPA axis reactivity test, main effects and interactions were examined with repeated measures ANOVA with rearing condition and supplier as between-subject factors and time as within-subject factor. Time-dependent *post hoc* tests were performed with Student’s paired t-test.

#### Non-parametric statistics

Main effects and interactions of longitudinal data sets were examined with the R package nparLD [59] with rearing condition and supplier as between-subject factors and time as within-subject factor. For this analysis, missing values in the alcohol access data set were imputed as the average of the flanking weeks for that individual, for all other analyses missing values were considered as missing. Time-dependent *post hoc* tests were performed with the Wilcoxon’s matched pairs test and group-dependent *post hoc* tests were performed with the Mann-Whitney U-test with continuity correction. The MCSF data were analyzed by rearing condition with the Mann-Whitney U-test with continuity correction and by estrus cycle stage with the Kruskal-Wallis ANOVA by ranks with *post hoc* Mann-Whitney U-test with continuity correction.

#### The trend analysis of MCSF data

In accordance with [38], parameters in the MCSF test can be grouped into functional categories, ranked and summed into compound parameters summarizing an animal’s performance in the test in five different behavioral categories: general activity, exploratory activity, risk assessment, risk taking and shelter seeking. The categories were analyzed identically to the other MCSF parameters.

## Results

### Experiment 1

The behavioral profile of the adult female MS15 and MS360 offspring (Sca; *n* = 15/group) was assessed with the MCSF test at 15 weeks of age. Directly after the behavioral test, the estrus cycle stage was determined by vaginal lavage.

#### MCSF performance by rearing condition

**Fig 2:**
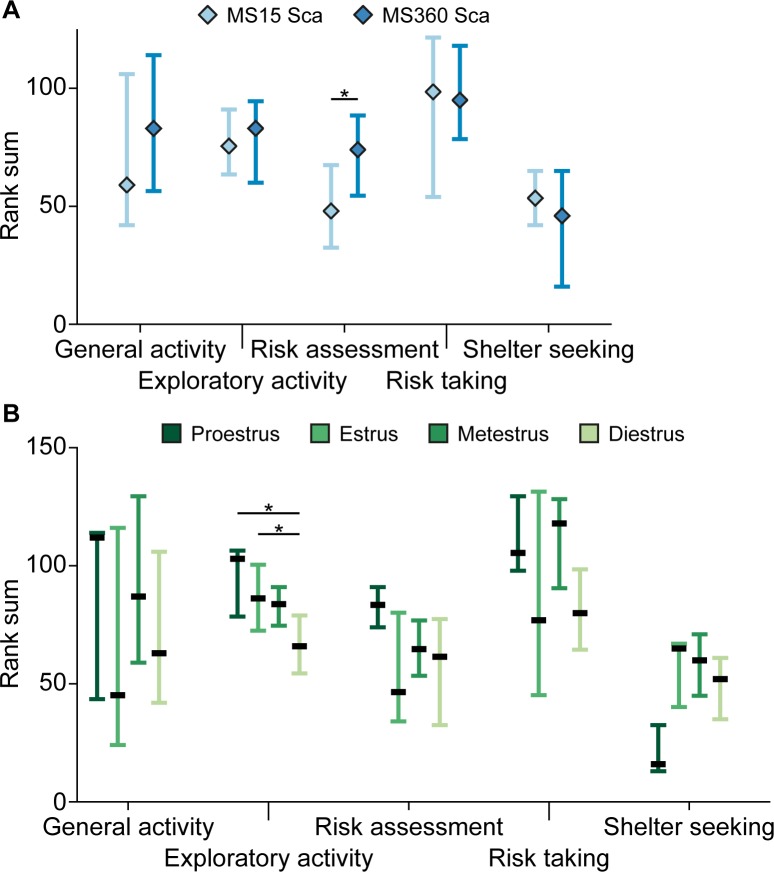
Trend analysis of the MCSF performance. Rankings of the functional behavioral categories in the trend analysis of the MCSF by A) rearing condition (n = 15/group) and B) estrus cycle stage (*n* = 3-11/group). Data are presented as group median with upper and lower quartiles. *p<0.05 with A) Mann-Whitney U-test and B) *post hoc* Mann-Whitney U-test after significant Kruskal-Wallis test.

The results from the MCSF test analyzed by rearing condition can be seen in S1 Table for the individual parameters and in Fig 2A for the trend analysis. None of the individual parameters showed any difference between the MS15 Sca and MS360 Sca groups. Analysis of the behavioral categories in the trend analysis revealed that MS360 Sca had higher risk assessment in the MCSF test than the MS15 Sca group (p<0.05).

At the time of testing, the body weights of MS15 Sca animals were 283.7±5.2 g and MS360 Sca animals weighed 271.3±6.2 (mean ± SEM), which was not significantly different according to Student’s unpaired t-test.

#### MCSF performance by estrus stage

The result for the MCSF test analyzed by estrus stage is shown in S2 Table and Fig 2B. Three animals were classified as in proestrus, six as estrus, eight as metestrus and eleven as diestrus. Two females had equally mixed cell types in their vaginal smears, and thus were these animals excluded from this analysis.

Four individual parameters showed a main effect of estrus stage: F bridge, D bridge, %D bridge and slope/bridge interval (p<0.05). Animals in diestrus had fewer visits to the bridge than animals in proestrus or estrus (p<0.01 and <0.05, respectively). Diestrus animals also spent less time on the bridge than animals in proestrus or metestrus measured both as absolute time (p<0.05 and <0.01, compared to pro- or metestrus respectively) and as percentages (p<0.05 and <0.01, respectively). Animals in proestrus had shorter delays between entering the bridge after first visit to the slope as shown by the higher slope/bridge interval, compared to animals in estrus, metestrus (p<0.05) and diestrus (p<0.01). Analysis of the behavioral categories in the trend analysis revealed a main effect of estrus cycle stage on exploratory activity (p<0.05) where diestrus animals had lower activity than both proestrus and estrus animals (p<0.05) (Fig 2B).

### Experiment 2

HPA axis activity and long-term voluntary alcohol intake were assessed in adult female MS15 and MS360 offspring (Rcc and Tac; *n* = 10/group, total *n* = 40 animals) starting at 10 weeks of age.

#### HPA axis reactivity

The HPA axis reactivity was assessed by exposing the animals to cat odor during 30 minutes of isolation. Blood samples for analysis of serum corticosterone levels were collected immediately before (baseline) and after (T0) the odor-exposure and 120 minutes (T120) later, after recovery in the home cage.

The protocol required serial removal of the animals from the home cage, and as this has been shown to induce an increase in corticosterone in adolescent animals [46], the effect of order was examined here as well. The corticosterone levels did not show any effect of order, neither when all three time-points were analyzed together nor when they were analyzed separately (for details see S1 Data).

**Fig 3:**
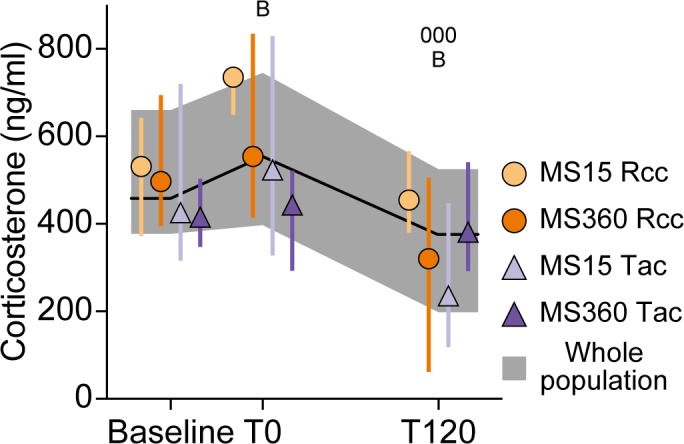
Corticosterone levels in serum during the HPA axis reactivity test. HPA axis reactivity testing of female MS15 and MS360 offspring from Rcc and Tac. Serum was collected for corticosterone analysis immediately before (baseline) and after (T0) cat-odor exposure during 30 minutes of isolation, and 120 min after recovery in the home cage (T120). Data are presented as medians with interquartile range for the whole population (*n* = 31–39), and by rearing condition × supplier (*n* = 7-10/group). B p<0.05 compared to baseline, 000 p<0.001 compared to T0 (*post hoc* Student’s paired t-test).

The result from the HPA axis reactivity test can be seen in Fig 3. There was a main effect of time (p<0.001), but no effects of rearing condition or supplier, and no interaction. The corticosterone levels increased from baseline to T0 (p<0.05) where after the levels decreased at T120 both compared to the baseline and T0 levels (p<0.05 and <0.001, respectively).

#### Voluntary alcohol intake: Two-bottle free-choice

**Fig 4:**
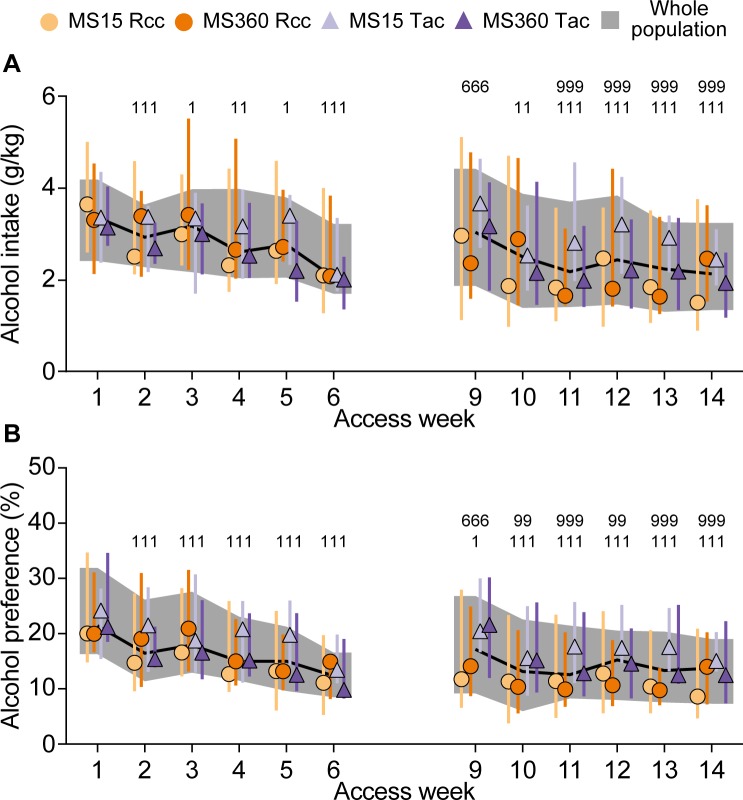
Alcohol intake and preference in the two-bottle free-choice paradigm. Alcohol A) intake and B) preference during the 14 weeks of alcohol access in the two-bottle free-choice (20% alcohol and water) paradigm on the modified intermittent access schedule (three days of alcohol, four days of water only), with an alcohol deprivation period week 7–8. The alcohol consumption was measured every 24 h during alcohol access, and averaged for the three access days in a week. Data are presented as medians with interquartile range for the whole population (*n* = 40), and by rearing condition × supplier (*n* = 10/group). 1 p<0.05, 11 <0.01, 111 <0.001 compared to week 1; 666 p<0.001 compared to week 6; 999 p< 0.001 compared to week 9 (*post hoc* Wilcoxon’s test).

The alcohol intake and preference during the 14 weeks of alcohol access in the two-bottle free-choice paradigm under the modified intermittent schedule is shown in Fig 4. During week 1–6, the animals had access to 20% alcohol and water, week 7 and 8 were alcohol deprivation weeks with no alcohol access, after which alcohol access was reinitiated for six additional weeks.

There was a main effect of time (p<0.001) for alcohol intake (Fig 4A) but no effect of rearing condition or supplier, and no interaction (see Table 2). The alcohol intake decreased from the second week of access and onwards, relative to week 1 (p<0.05 for week 3 and 5; p<0.01 for week 4 and 10; and p<0.001 for week 2, 6 and 11–14), except for week 9. After the alcohol deprivation period, the alcohol intake increased at week 9 compared to week 6 (p<0.001), indicating an alcohol-deprivation effect. Thereafter, the intake decreased to levels comparable to week 6, and from week 11 and onwards the alcohol intake was lower than at week 9 (p<0.001).

**Table 2:** Statistical main effects and interactions during the long-term voluntary alcohol intake.

Parameters		Main effects	Interactions
**Two-bottle choice**			
	Intake	Time***	-
	Preference	Time***	-
**Three-bottle choice**			
	Total intake	Time **	Time × Supplier*
	5% intake	Time*	-
	20% intake	Time***, Supplier*	-
	Total preference	-	Time × Supplier x RC*
	5% preference	-	-
	20% preference	Time**, Supplier**	-
**Water intake**		Time***, Supplier*	-
**Total fluid intake**		Time***, Supplier*	Time × Supplier***

Main effects and interactions of time, supplier and rearing condition (RC), during the long-term voluntary alcohol intake with two- or three-bottle free-choice paradigms in the modified intermittent schedule during 20 weeks.

*p<0.05

**p<0.01

***p<0.001 in the R package nparLD.

The alcohol preference (Fig 4B) also showed a main effect of time (p<0.001) with no involvement of rearing condition or supplier (see Table 2). The preference decreased from week 2 and onwards relative to week 1 (p<0.05 for week 9 and p<0.001 for week 2–6 and 10–14). The alcohol deprivation effect was evident here as well, with an increased preference week 9 compared to week 6 (p<0.001). The effect dissipated the following weeks, as the preference week 10–14 did not differ from week 6 but showed a decrease compared to week 9 (p<0.01 for week 10 and 12; p<0.001 for week 11 and 13–14).

#### Voluntary alcohol intake: Three-bottle free-choice

**Fig 5:**
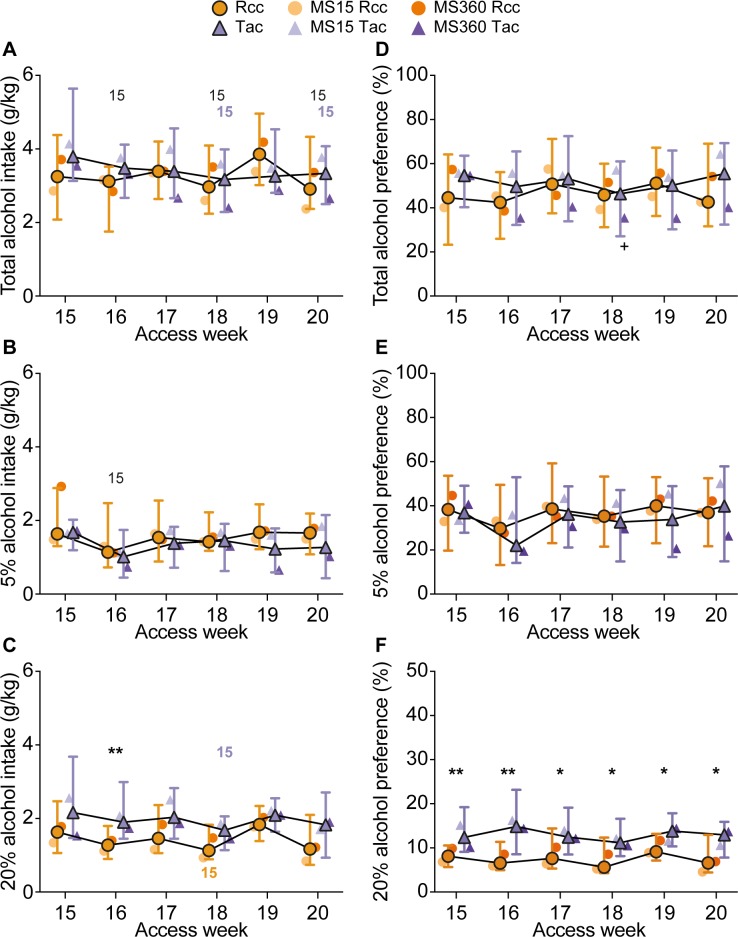
Alcohol intake and preference in the three-bottle free-choice paradigm. Alcohol A-C) intake and D-F) preference during the three-bottle free-choice paradigm. The animals had access to 5% (B, E) and 20% (C, F) alcohol and water three consecutive days per week for six weeks. Data are presented as weekly averages with supplier medians (the unit of analysis, n = 20/group) with interquartile range and median by supplier × rearing condition (n = 10/group). * p < 0.05, ** < 0.01 comparing Rcc and Tac animals (post hoc Mann-Whitney U-test); 15 (black) p < 0.05 compared to whole population level at week 15, 15 (orange) < 0.05 compared to Rcc level at week 15, 15 (purple) < 0.05 compared to Tac level at week 15, + < 0.05 comparing MS360 Tac females with its week 15 value (post hoc Wilcoxon’s test).

During alcohol access week 15–20, the animals had access to 5% alcohol in addition to the 20% alcohol and water. The results of analysis of main effects and interactions are summarized in Table 2, and the 5%, 20% and total alcohol intake and preference are shown in Fig 5.

The alcohol intake showed main effects of time for all three measures in addition to an effect of supplier for 20% intake, and an interaction between time and supplier for the total intake (for p-values see Table 2). The total alcohol intake (Fig 5A) did not reveal any supplier-dependent differences within the individual time points, but Tac animals showed a decrease in total intake at weeks 18 and 20 compared to week 15 (p<0.05). Rcc animals did not exhibit any changes over time, while the population as a whole showed decreased total alcohol intake at weeks 16, 18 and 20 compared to week 15 (p<0.05). For the whole population, only a minor time-dependent difference was observed for the 5% alcohol intake (Fig 5B), as it decreased from week 15 to week 16 (p<0.05) and then remained stable. For the 20% alcohol intake (Fig 5C), a supplier-dependent difference was detected, as Tac animals had higher 20% intake than Rcc animals at week 16. Over time, the 20% intake was stable, except for week 18 where the intake was decreased in both suppliers compared to the respective intake at week 15 (p<0.05).

The total and 5% alcohol preferences did not show any main effects, but for total preference, there was a three-way interaction between time, supplier and rearing condition; 20% preference had main effects of time and supplier (for p-values see Table 2). *Post hoc* analysis of the total alcohol preference (Fig 5D) did not reveal any differences independent of level of analysis, except for week 18, were MS360 Tac had a decreased preference compared to week 15. No group- or time-dependent differences were found for 5% alcohol preference (Fig 5E). For 20% alcohol preference (Fig 5F), there were no time-dependent differences, however, Tac animals had higher preference for 20% alcohol than the Rcc animals over the whole period (p<0.01 week 15–16; p<0.05 week 17–20).

#### Water and total fluid intake

Both water and total fluid intake showed main effects of time and supplier, while only total fluid intake showed an interaction between time and supplier (for p-values see Table 2). As shown in detail in S2 Fig, Tac animals had lower water intake than Rcc animals during five weeks of the two-bottle free-choice period. However, during the three-bottle free-choice period there was no difference in water consumption between rats from the two suppliers. The total fluid intake showed more supplier-dependent differences; during the two-bottle free-choice period, Tac animals had lower fluid consumption than Rcc animals on seven of the weeks and on five of the weeks during the three-bottle free-choice period.

#### Fecal corticosterone

Fecal corticosterone was assessed as a measure of corticosterone load with and without alcohol access. Samples were collected after the three days of alcohol access in week 19; when the animals had access to 5% and 20% alcohol and water; and after four days of water only after last alcohol access in week 20.

**Fig 6:**
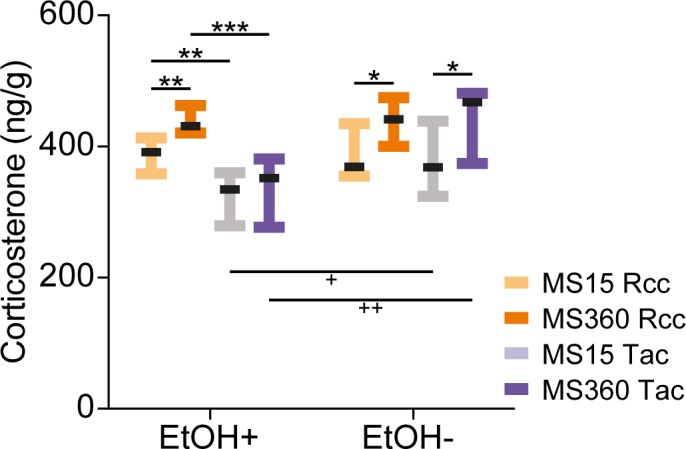
Corticosterone levels in feces with and without alcohol access. Fecal corticosterone was measured directly after three days of alcohol access (EtOH+) and after four days of water only (EtOH-). Data are presented as group medians with interquartile range (*n* = 10/group). *p<0.05, **<0.01, ***<0.001 (*post hoc* Mann-Whitney U-test), +p<0.05, ++<0.01 (*post hoc* Wilcoxon’s test).

There were main effects of rearing condition, supplier and alcohol (+/-) (p<0.001) as well as an interaction between supplier and alcohol (+/-) (p<0.001). When sampled directly after alcohol access (+), MS360 Rcc animals had higher levels of fecal corticosterone compared to MS15 Rcc animals (p<0.01) (Fig 6). In the Tac animals, there was no difference between the rearing conditions during alcohol access (+), but both MS15 and MS360 Tac animals had lower levels of fecal corticosterone than their respective Rcc counterpart (p<0.01 and <0.001, respectively). When sampled without alcohol access (-), both MS360 groups had higher fecal corticosterone levels than their respective MS15 group (p<0.05), although no supplier-dependent difference was found. When comparing the fecal corticosterone levels with and without alcohol access, it was revealed that Tac animals, independent of rearing condition, had higher levels when alcohol was not present than when alcohol was present (p<0.05 for MS15 Tac and p<0.01 for MS360 Tac animals), while no effect dependent on alcohol access (+/-) was observed in Rcc animals.

#### Adult body weights

The body weights of the animals were assessed after the last blood sample of the HPA axis reactivity test and after the last alcohol access period on the weeks of alcohol access. The repeated measures ANOVA showed a main effect of time (p<0.001) but no effect or interaction with rearing condition or supplier. For the whole population, the body weight increased continuously until the introduction of the second bottle of alcohol at alcohol access week 15, when it decreased slightly before recovering at week 19 (for p-values see S3 Table).

## Discussion

Daily maternal separation, for 15 or 360 min during PNDs 1–21, were used to model safe *versus* adverse rearing conditions. In adulthood, the female offspring were assessed for differences in behavior, HPA axis activity and voluntary alcohol intake. The present study demonstrates few long-term consequences of prolonged MS in adult female offspring. Instead, the observed differences are more dependent on estrus cycle stage or by the choice of animal supplier.

In the MCSF test, used to assess the behavioral profile of the adult female offspring, there were no differences between MS15 and MS360 among the individual parameters, but with the use of the trend analysis, a difference in risk assessment was revealed, where MS360 Sca females had higher risk assessment than MS15 Sca females. That a difference only emerged in the trend analysis, when parameters are grouped into functional categories, speaks to the added value of the trend analysis and the rank-based summation of parameters with related functionality [38]. Previously, male offspring reared using the same MS paradigm, have been shown to have more differences among individual parameters associated with exploratory activity, risk taking and risk assessment. MS360 males had higher exploration and altered risk-taking and risk-assessing profiles than standard reared males, while MS15 males constituted an intermediate group [25].

When the females in the present study instead were divided by estrus cycle stage, more pronounced differences emerged. Among the individual parameters, differences in risk-taking and impulsive-like behaviors were discovered and the trend analysis showed an effect on exploratory activity. Females in diestrus had lower scores in parameters associated with risk taking on the bridge and they had a lower exploratory activity in the trend analysis; proestrus females on the other hand showed increased impulsive-like behavior. It is well recognized that females in estrus display increased activity, evident by enhanced wheel-running activity [60,61]. It has however been suggested that wheel-running activity is not an exploratory behavior and does not involve explorative strategies [60]. One interesting finding herein was that the females displayed similar general activity independent of estrus cycle stage. This finding is congruent with a previous study of female behavioral profiles in the MCSF test, in which no difference in measures of general activity were detected between females in estrus and females in the other stages of the estrus cycle [39]. It has been suggested that several tests used in behavioral neuroscience are “artificial”, with environments poor of stimuli, and that environments that are more complex trigger exploratory behavior [60]. With regard to complexity, the multivariate and ethologically founded design of the MCSF [39] can be an advantage, suggested by the capture of the fine, but important, difference between general and exploratory activity. Furthermore, the MCSF test has a greater range of sensitivity than, for instance, the elevated plus maze and the open field tests [17,23,25,62]. That more differences were revealed based on estrus cycle stage than on rearing condition was unexpected, since it previously has been shown that females are less variable than males, in the MCSF in particular [39] and in behavioral tests in general [34]. Taken together, the finding that the rearing-dependent differences are less pronounced than estrus cycle-dependent variations support the notion that adult female behavior is not highly influenced by early-life experiences. Finally, it would have been highly interesting to compare estrus cycle stage dependent on rearing condition, but the distribution between the different estrus cycle stages was skewed, rendering such stratification impossible due to low power. On that account, the comparisons herein with the proestrus group should be interpreted with caution as there are only three individuals classified into that estrus cycle stage.

Additional studies have examined sex differences in response to MS with other behavioral paradigms. For instance, twice daily MS for 180 min, decreased the latency to emerge from the tube and explore the arena in the defensive withdrawal test, compared to both standard reared and twice daily MS15 female rats, while no rearing-dependent differences were observed in male rats [63]. Moreover, 180 min of MS during PNDs 2–14 resulted in less pronounced effects on baseline startle amplitude in female than in male rats, relative to the respective standard reared controls. It also decreased light-potentiation of startle in females, indicating reduced anxiety-like behavior in female rats [64]. In another study, individual MS15 with handling and individual MS180 during PNDs 2–21 were compared with a non-handled group. In adulthood, handled male rats spent significantly more time in the open arms of the elevated plus maze than non-handled male rats, while both female groups, i.e. handled and MS180 rats, spent more time in the open arms than non-handled female rats [65]. Another study, comparing the effects of 270 min MS and standard reared animals revealed that adult male MS270 rats had higher ambulation in the open field test than female MS rats, while no difference between MS270 and standard rearing was observed within each sex [66]. Finally, in females, 180 min of MS on PNDs 2–14 did not have any effect on the performance in the forced swim or open field tests compared to standard reared controls [30]. These studies illustrate the wide variety in MS protocols used with differences in e.g. separation durations, number of separation days and choice of control group or groups. However, regardless of the methodological differences these studies come to similar conclusions regarding differences in long-term behavioral effects of early-life rearing conditions, with females showing less pronounced rearing-dependent effects than males.

In the HPA axis reactivity test, adult females exhibited a typical reactivity with increased corticosterone levels after challenge and levels returning below baseline after recovery. However, no effect of rearing condition or supplier was found which is in line with findings in adolescent females [46]. A lack of rearing-dependent effect contrasts some studies in adult males, where prolonged separation has revealed either blunted [25] or elevated [67] levels of stress-induced corticosterone relative to short MS. However, no effect of rearing condition on adult male HPA axis reactivity has also been reported [68,69]. This mirrors the human literature, where studies also show varied effects on HPA axis activity after chronic stress [70]. HPA axis reactivity in Wistar rats of different supplier origin, have to our knowledge, not been compared before in adult animals, but adolescent animals did not show any effect of supplier, independent of sex, after MS15 or MS360 [46].

In the present study, adult MS15 and MS360 females did not differ in their alcohol intake, even after prolonged access or in different free-choice intake paradigms. In the last alcohol access period, under the three-bottle free-choice paradigm, an interaction with rearing condition was present but no rearing-dependent differences were revealed, except for one time-dependent difference for MS360 Tac females. No rearing-dependent effect on alcohol intake in female animals is in agreement with previous studies in Wistar rats comparing MS360 to MS15, and standard rearing [31,33], MS240 compared to handled [71] and in alcohol-preferring rats subjected to MS360 relative to MS15 [32], using continuous access paradigms with lower alcohol concentrations than the present study. In contrast, the majority of studies in males have shown that prolonged MS results in increased alcohol intake compared to short MS or standard reared males, using both intermittent and continuous alcohol access paradigms, even though there are reports of no rearing-dependent differences between males as well, this has been summarized in several reviews [10,72,73].

In this study, female Rcc and Tac animals acquired and maintained alcohol intake on a stable level over time. In addition, no supplier-dependent differences were seen when the animals had the choice of one alcohol concentration (20%) and water, but a difference emerged when a second alcohol concentration (5%) was added to the paradigm. Tac animals had consistently higher preference for 20% alcohol in the three-bottle free-choice paradigm than the Rcc animals, while the total alcohol preference did not differ. This supplier-dependent effect is puzzling; the few studies that have examined supplier-dependent effects on alcohol intake have done so in male, standard reared animals, and Rcc animals have consistently had higher alcohol intake and preference than Tac animals in intermittent access paradigms [41,43]. Additionally, a clear sub-group of high drinking animals in Rcc males has been described [41,49], and there is no such grouping among Rcc females in the present study. If the findings herein are indicative of true sex-dependent effects on supplier-dependent differences, or if it is influenced by the rearing conditions and/or previous experimental procedure herein is impossible to say and requires further investigation before any conclusion can be drawn. Additionally, the underlying causes for supplier-dependent differences are unexplored. The Wistar sub-strains available today have been generated and maintained through complex derivations [45]. Although external factors, such as different husbandry conditions, are believed to play an important role [74], genetic drift likely has a greater impact. For instance, it was recently reported that Wistar rats from certain suppliers carry a stop codon resulting in loss of mGlu2 receptor function in different frequencies [45], which highlight the importance of further investigation of supplier-specific effects.

The only measure that showed a robust effect of rearing condition in this study was the corticosterone load, as measured by fecal corticosterone excretion. MS360 animals had higher levels of fecal corticosterone than the respective MS15 groups, except for Tac animals when alcohol was present, where no rearing-dependent difference was evident. Tac animals were also the only ones to show an increase in fecal corticosterone when alcohol access was withdrawn compared to when alcohol was present. The effect of alcohol access can be either an intrinsic supplier-dependent effect or an effect of the increased preference of 20% alcohol in the Tac animals. The rearing-dependent effect is anyhow highly interesting since it is the only exception to the female resilience seen otherwise in this study. That this difference was not detected under basal conditions while present after long-term perturbation (i.e. the alcohol access with single-housing) might be evidence of increased buffering capabilities in females compared to males, in line with the multiple-hit concept of adaptation after early-life adversity [9]. This concept involves different combinations of genes, early environment and later challenges in the development of either vulnerability or resilience after early adversity [9].

The present study uses a MS model comparing an adverse, prolonged separation condition with a protective, short separation. These rearing conditions have exhibited the most stable rearing condition-dependent differences when used in male animals, relative to the use of other types of control groups [17,75]. That there were few long-term differences between the two rearing conditions used herein further strengthens the results from previous studies regarding female animals’ low sensitivity to these types of manipulations of the early-life environment. However, what needs further studies is the result regarding the corticosterone load, to our knowledge reported for the first time herein. It is important to bear in mind that this result was obtained using MS360 relative to MS15 and the result may benefit from corroboration with other rearing conditions and possibly other models of early-life experiences.

The present experiments add important pieces of information to the set of studies investigating the long-term effects of MS15 and MS360 on risk and protective factors for behavioral and hormonal alterations in addition to vulnerability to excessive alcohol intake in female rats. It is well acknowledged that the vulnerability for, and severity of, several psychopathological conditions display differences between males and females, in humans as well as in experimental animals [11,12]. Why MS seems to result in different outcomes for male and female rats requires further investigation. While it is tempting to speculate on subjects of sex-dependent differences including developmental, hormonal, behavioral and genetic differences, there is simply not enough studies investigating this matter. It may also be that the early-life rearing conditions have minor consequences for basal functioning in female rats. Thus, additional challenges or stimuli occurring later in life may reveal differences, as seen herein with the corticosterone load after single housing and long-term alcohol access. The MS model may represent one of the experimental tools in the search for genetic and environmental interactions involved in risk and protective factors and subsequent adult phenotypes.

## Supporting information

S1 Data (XLSX)

S1 Fig Schematic layout of the MCSF arena. The arena (70×70 cm) is divided into zones by walls (solid, black lines) or imagined boundaries (dashed lines): 1, the central circle (CTRCI); 2, the center; 3a-c, corridors; 4, the dark corner room (DCR); 5, the hurdle with hole-board for nose poking; 6, the slope, leading up to the brightly lit bridge (7). Zone 1 and 7 are considered as risk areas, zone 6 together with the manually scored behavior SAP (stretched attend posture) are associated with risk assessment, zones 2 and 3 are transit zones generating measures of activity and exploration, zone 4 is a shelter and zone 5 is considered as an exploratory incentive. The saturation levels in the figure reflect the illumination levels in the arena.
(EPS)

S2 Fig Water and fluid intake during the long-term voluntary alcohol intake. A) Water and B) total fluid intake during the 20 weeks of long-term voluntary alcohol intake in Experiment 2. The arrows indicate the week where the third bottle (5% alcohol) was added to the drinking paradigm. Data are presented as medians by supplier (*n* = 20/group) with interquartile range, and medians by rearing condition × supplier (*n* = 10/group). *p<0.05, **<0.01, ***<0.001 comparing the suppliers with Mann-Whitney U-test. 1, 6, 9, 14, 15 = p<0.05 with Wilcoxon’s test comparing subsequent weeks with week 1, 6, 9, 14 and 15, respectively. Orange number signifies statistically significant differences within the Rcc animals and purple numbers differences within Tac animals. Different levels of significance below p<0.05 are not differentiated for the time-dependent differences.
(EPS)

S1 Table Individual parameters of the MCSF test by rearing condition. Results from the multivariate concentric square field™ (MCSF) test in MS15 Sca and MS360 Sca rats (*n* = 15/group) in Experiment 1. Behavioral parameters recorded during the 20-min trial of the MCSF test. Values represent median with interquartile range. No statistically significant difference was discovered between the groups according to the Mann-Whitney U-test. *Abbreviations*: CTRCI, central circle; DCR, dark corner room; D, duration; F, frequency; L, latency; SAP, stretched attend posture; TOTACT, total activity; TOTCORR, total corridor.
(DOCX)

S2 Table Individual parameters of the MCSF test by estrus cycle stage. Results from the multivariate concentric square field™ (MCSF) test in animals divided by estrus cycle stage: proestrus (P, *n* = 3), estrus (E, *n* = 6), metestrus (M, *n* = 8) and diestrus (D, *n* = 11) in Experiment 1. Behavioral parameters recorded during the 20-min trial of the MCSF test. Values represent median with interquartile range. *p<0.05, **<0.01 with *post hoc* Mann-Whitney U-test after p<0.05 with Kruskal-Wallis test. *Abbreviations*: CTRCI, central circle; DCR, dark corner room; D, duration; F, frequency; L, latency; SAP, stretched attend posture; TOTACT, total activity; TOTCORR, total corridor.
(DOCX)

S3 Table Body weights of animals in Experiment 2. Body weights (g) of MS15 and MS360 Rcc/Tac animals throughout Experiment 2 (*n* = 10/group, 40 total). Values represent mean and standard error of the mean (SEM). Six reference time points (HPA, week 1, 6, 9, 14 and 15) were used to compare all subsequent time points. *p<0.05, ***<0.001 *post hoc* Student’s t-test on whole population after significant main effect of time (p<0.001, repeated measures ANOVA).
(DOCX)
