# Supplementary material for: Few long-term consequences after prolonged maternal separation in female Wistar rats
Source: PLoS One. 2017 Dec 21;12(12):e0190042. doi: 10.1371/journal.pone.0190042 (PMC5739456; doi:10.1371/journal.pone.0190042)
Supplement: S1 Table — Results from the multivariate concentric square field™ (MCSF) test in MS15 Sca and MS360 Sca rats (n = 15/group) in Experiment 1. Behavioral parameters recorded during the 20-min trial of the MCSF test. Values represent median with interquartile range. No statistically significant difference was discovered between the groups according to the Mann-Whitney U-test. Abbreviations: CTRCI, central circle; DCR, dark corner room; D, duration; F, frequency; L, latency; SAP, stretched attend posture; TOTACT, total activity; TOTCORR, total corridor. (DOCX) [file pone.0190042.s004.docx]

**Table S1. Individual parameters of the MCSF test by rearing condition.**

| **Functional category** | **Parameter** | **MS15 Sca** | | | **MS360 Sca** | | |
| --- | --- | --- | --- | --- | --- | --- | --- |
|  |  | Median | Quartiles | | Median | Quartiles | |
| General activity | TOTACT | 77.0 | 66.0 - | 100.0 | 91.0 | 79.0 - | 107.0 |
|  | F TOTCORR | 30.0 | 26.0 - | 41.0 | 36.0 | 30.0 - | 45.0 |
|  | %F TOTCORR | 40.0 | 39.4 - | 43.1 | 40.2 | 38.0 - | 43.0 |
|  | F center | 21.0 | 18.0 - | 27.0 | 23.0 | 20.0 - | 29.0 |
|  | D center | 237.7 | 222.7 - | 296.6 | 201.4 | 173.5 - | 242.8 |
|  | D/F center | 10.6 | 9.4 - | 11.9 | 9.0 | 7.2 - | 11.5 |
|  | %F center | 27.3 | 25.2 - | 30.1 | 25.6 | 23.5 - | 28.0 |
|  | %D center | 19.8 | 18.6 - | 24.7 | 16.8 | 14.5 - | 20.2 |
|  | Distance | 4065.2 | 3989.3 - | 4728.7 | 4370.8 | 4052.1 - | 4839.1 |
|  | Velocity | 3.4 | 3.3 - | 4.1 | 3.8 | 3.4 - | 4.1 |
| Exploratory activity | L leave | 25.7 | 19.2 - | 51.6 | 26.8 | 14.2 - | 46.6 |
|  | D TOTCORR | 388.6 | 314.6 - | 453.9 | 437.8 | 349.7 - | 489.2 |
|  | D/F TOTCORR | 11.4 | 9.5 - | 13.1 | 11.9 | 8.7 - | 15.8 |
|  | %D TOTCORR | 32.4 | 26.2 - | 37.8 | 36.5 | 29.1 - | 40.8 |
|  | L hurdle | 217.0 | 168.7 - | 298.8 | 216.9 | 130.6 - | 404.4 |
|  | F hurdle | 4.0 | 3.0 - | 5.0 | 5.0 | 3.0 - | 6.0 |
|  | D hurdle | 82.2 | 59.4 - | 113.5 | 104.5 | 88.7 - | 125.0 |
|  | D/F hurdle | 21.3 | 13.7 - | 29.3 | 21.5 | 17.7 - | 31.1 |
|  | %F hurdle | 5.2 | 4.3 - | 6.0 | 5.3 | 3.8 - | 6.2 |
|  | %D hurdle | 6.9 | 5.0 - | 9.5 | 8.7 | 7.4 - | 10.4 |
|  | Nose pokes | 6.0 | 4.0 - | 9.0 | 7.0 | 3.0 - | 12.0 |
|  | Rearing | 31.0 | 26.0 - | 33.0 | 28.0 | 22.0 - | 36.0 |
| Risk assessment | L slope | 202.3 | 132.2 - | 339.4 | 146.7 | 95.9 - | 484.8 |
|  | F slope | 6.0 | 5.0 - | 8.0 | 8.0 | 7.0 - | 10.0 |
|  | D slope | 74.2 | 54.8 - | 102.4 | 96.0 | 67.7 - | 119.7 |
|  | D/F slope | 11.6 | 8.2 - | 16.8 | 11.4 | 9.0 - | 18.1 |
|  | %F slope | 8.0 | 7.0 - | 9.9 | 9.6 | 7.9 - | 11.3 |
|  | %D slope | 6.2 | 4.6 - | 8.5 | 8.0 | 5.6 - | 10.0 |
|  | SAP DCR | 0.0 | 0.0 - | 1.0 | 1.0 | 0.0 - | 2.0 |
|  | SAP hurdle | 0.0 | 0.0 - | 0.0 | 0.0 | 0.0 - | 0.0 |
|  | SAP bridge | 0.0 | 0.0 - | 0.0 | 0.0 | 0.0 - | 0.0 |
|  | SAP total | 0.0 | 0.0 - | 1.0 | 1.0 | 0.0 - | 3.0 |
| Risk taking | L CTRCI | 169.5 | 46.0 - | 273.8 | 127.5 | 54.1 - | 278.2 |
|  | F CTRCI | 5.0 | 3.0 - | 6.0 | 5.0 | 4.0 - | 6.0 |
|  | D CTRCI | 6.0 | 2.7 - | 9.6 | 5.8 | 3.9 - | 7.5 |
|  | D/F CTRCI | 1.1 | 1.0 - | 1.4 | 1.0 | 0.9 - | 1.3 |
|  | %F CTRCI | 5.4 | 4.3 - | 7.6 | 5.0 | 4.1 - | 7.3 |
|  | %D CTRCI | 0.5 | 0.2 - | 0.8 | 0.5 | 0.3 - | 0.6 |
|  | L bridge | 222.4 | 143.3 - | 363.8 | 177.1 | 105.9 - | 478.5 |
|  | F bridge | 3.0 | 3.0 - | 4.0 | 4.0 | 3.0 - | 5.0 |
|  | D bridge | 115.8 | 82.5 - | 146.2 | 139.9 | 100.9 - | 162.8 |
|  | D/F bridge | 30.3 | 27.5 - | 43.8 | 34.3 | 32.6 - | 40.0 |
|  | %F bridge | 4.3 | 2.8 - | 4.7 | 4.9 | 3.6 - | 6.3 |
|  | %D bridge | 9.7 | 6.9 - | 12.2 | 11.7 | 8.4 - | 13.6 |
| Shelter seeking | L DCR | 99.5 | 63.2 - | 182.3 | 144.0 | 49.0 - | 162.0 |
|  | F DCR | 6.0 | 5.0 - | 10.0 | 6.0 | 5.0 - | 11.0 |
|  | D DCR | 225.8 | 151.7 - | 272.8 | 163.0 | 71.5 - | 251.4 |
|  | D/F DCR | 25.1 | 20.6 - | 45.5 | 22.6 | 17.9 - | 27.2 |
|  | %F DCR | 8.9 | 7.8 - | 10.0 | 7.6 | 6.4 - | 10.0 |
|  | %D DCR | 18.8 | 12.6 - | 22.7 | 13.6 | 6.0 - | 21.0 |
| Anxiety-like behavior | F risk/shelter index | -0.33 | -0.54 - | -0.27 | -0.29 | -0.47 - | -0.09 |
|  | D risk/shelter index | -0.28 | -0.55 - | -0.03 | -0.13 | -0.34 - | 0.10 |
| Impulsive-like behavior | Slope/bridge interval | -0.06 | -0.08 - | -0.05 | -0.06 | -0.31 - | -0.05 |
| Other | Grooming | 2.0 | 1.0 - | 3.0 | 1.0 | 0.0 - | 2.0 |
|  | Urine | 5.0 | 2.0 - | 6.0 | 3.0 | 2.0 - | 6.0 |
|  | Boli | 0.0 | 0.0 - | 0.0 | 0.0 | 0.0 - | 0.0 |

Results from the multivariate concentric square field™ (MCSF) test in MS15 Sca and MS360 Sca rats (*n* = 15/group) in Experiment 1. Behavioral parameters recorded during the 20-min trial of the MCSF test. Values represent median with interquartile range. No statistically significant difference was discovered between the groups according to the Mann-Whitney U-test. *Abbreviations:* CTRCI, central circle; DCR, dark corner room; D, duration; F, frequency; L, latency; SAP, stretched attend posture; TOTACT, total activity; TOTCORR, total corridor.
