# Supplementary material for: Few long-term consequences after prolonged maternal separation in female Wistar rats
Source: PLoS One. 2017 Dec 21;12(12):e0190042. doi: 10.1371/journal.pone.0190042 (PMC5739456; doi:10.1371/journal.pone.0190042)
Supplement: S2 Table — Results from the multivariate concentric square field™ (MCSF) test in animals divided by estrus cycle stage: proestrus (P, n = 3), estrus (E, n = 6), metestrus (M, n = 8) and diestrus (D, n = 11) in Experiment 1. Behavioral parameters recorded during the 20-min trial of the MCSF test. Values represent median with interquartile range. *p<0.05, **<0.01 with post hoc Mann-Whitney U-test after p<0.05 with Kruskal-Wallis test. Abbreviations: CTRCI, central circle; DCR, dark corner room; D, duration; F, frequency; L, latency; SAP, stretched attend posture; TOTACT, total activity; TOTCORR, total corridor. (DOCX) [file pone.0190042.s005.docx]

**Table S2. Individual parameters of the MCSF test by estrus cycle stage.**

| **Functional category** | **Parameter** | **Proestrus** | | | **Estrus** | | | **Metestrus** | | | **Diestrus** | | | **P value** |
| --- | --- | --- | --- | --- | --- | --- | --- | --- | --- | --- | --- | --- | --- | --- |
|  |  | Median | Quartiles | | Median | Quartiles | | Median | Quartiles | | Median | Quartiles | |  |
| General activity | TOTACT | 98.0 | 73.0 - | 108.0 | 68.0 | 65.0 - | 101.0 | 92.5 | 80.0 - | 109.0 | 77.0 | 70.0 - | 100.0 |  |
|  | F TOTCORR | 40.0 | 28.0 - | 41.0 | 26.5 | 24.0 - | 41.0 | 39.5 | 30.5 - | 45.5 | 34.0 | 27.0 - | 41.0 |  |
|  | %F TOTCORR | 38.4 | 38.0 - | 40.8 | 39.8 | 38.5 - | 40.6 | 39.2 | 37.7 - | 42.4 | 42.6 | 39.4 - | 43.6 |  |
|  | F center | 23.0 | 21.0 - | 31.0 | 18.0 | 16.0 - | 26.0 | 24.5 | 21.0 - | 27.0 | 21.0 | 20.0 - | 29.0 |  |
|  | D center | 178.2 | 173.5 - | 224.1 | 204.0 | 190.6 - | 257.6 | 239.9 | 186.2 - | 261.2 | 227.0 | 222.7 - | 358.2 |  |
|  | D/F center | 7.5 | 7.2 - | 8.5 | 10.5 | 9.2 - | 11.9 | 9.0 | 8.0 - | 10.4 | 10.8 | 9.7 - | 14.7 |  |
|  | %F center | 28.7 | 23.5 - | 28.8 | 25.7 | 25.2 - | 26.0 | 25.3 | 23.9 - | 27.9 | 28.2 | 23.9 - | 30.1 |  |
|  | %D center | 14.9 | 14.5 - | 18.7 | 17.0 | 15.9 - | 21.5 | 20.0 | 15.5 - | 21.8 | 18.9 | 18.6 - | 29.9 |  |
|  | Distance | 4913.9 | 4052.1 - | 5321.3 | 4189.6 | 3565.3 - | 4481.6 | 4223.0 | 4029.3 - | 4944.3 | 4107.6 | 3989.3 - | 4727.7 |  |
|  | Velocity | 4.1 | 3.4 - | 4.5 | 3.5 | 3.0 - | 3.8 | 3.8 | 3.4 - | 4.2 | 3.4 | 3.3 - | 4.0 |  |
| Exploratory activity | L leave | 14.2 | 7.9 - | 16.3 | 35.3 | 25.7 - | 45.9 | 21.5 | 10.6 - | 39.5 | 37.6 | 19.2 - | 66.8 |  |
|  | D TOTCORR | 488.5 | 265.7 - | 517.6 | 336.1 | 273.2 - | 373.2 | 391.7 | 358.7 - | 455.7 | 444.6 | 346.4 - | 468.5 |  |
|  | D/F TOTCORR | 12.6 | 6.6 - | 17.4 | 11.1 | 8.7 - | 11.4 | 9.9 | 8.7 - | 13.8 | 12.4 | 10.2 - | 13.4 |  |
|  | %D TOTCORR | 40.7 | 22.1 - | 43.1 | 28.0 | 22.8 - | 31.1 | 32.6 | 29.9 - | 38.0 | 37.1 | 28.9 - | 39.0 |  |
|  | L hurdle | 216.9 | 74.8 - | 335.5 | 147.2 | 50.7 - | 378.8 | 269.6 | 145.5 - | 408.5 | 217.0 | 195.9 - | 324.8 |  |
|  | F hurdle | 6.0 | 3.0 - | 8.0 | 4.0 | 3.0 - | 6.0 | 5.0 | 3.0 - | 6.0 | 4.0 | 3.0 - | 5.0 |  |
|  | D hurdle | 131.0 | 104.5 - | 135.0 | 95.0 | 54.6 - | 112.0 | 105.6 | 86.2 - | 112.5 | 82.2 | 58.9 - | 113.5 |  |
|  | D/F hurdle | 21.8 | 13.1 - | 45.0 | 20.8 | 12.5 - | 30.1 | 19.9 | 17.6 - | 30.4 | 21.3 | 19.6 - | 29.8 |  |
|  | %F hurdle | 5.6 | 4.1 - | 8.2 | 6.0 | 5.7 - | 6.1 | 4.9 | 3.8 - | 5.5 | 4.3 | 3.7 - | 5.7 |  |
|  | %D hurdle | 10.9 | 8.7 - | 11.3 | 7.9 | 4.6 - | 9.3 | 8.8 | 7.2 - | 9.4 | 6.9 | 4.9 - | 9.5 |  |
|  | Nose pokes | 10.0 | 2.0 - | 15.0 | 9.0 | 6.0 - | 14.0 | 4.5 | 3.5 - | 10.0 | 5.0 | 3.0 - | 7.0 |  |
|  | Rearing | 34.0 | 21.0 - | 53.0 | 28.5 | 27.0 - | 36.0 | 29.5 | 22.5 - | 37.5 | 31.0 | 26.0 - | 33.0 |  |
| Risk assessment | L slope | 257.5 | 103.6 - | 514.7 | 111.1 | 84.6 - | 189.5 | 219.9 | 99.5 - | 415.1 | 299.5 | 143.9 - | 393.1 |  |
|  | F slope | 9.0 | 7.0 - | 12.0 | 7.0 | 6.0 - | 8.0 | 8.5 | 6.0 - | 12.5 | 7.0 | 5.0 - | 8.0 |  |
|  | D slope | 119.7 | 96.0 - | 126.6 | 67.7 | 60.2 - | 102.4 | 89.2 | 82.1 - | 101.3 | 63.1 | 54.8 - | 101.5 |  |
|  | D/F slope | 13.3 | 8.0 - | 18.1 | 10.3 | 7.5 - | 20.4 | 10.5 | 8.6 - | 14.2 | 11.0 | 8.2 - | 16.8 |  |
|  | %F slope | 9.6 | 9.2 - | 11.1 | 8.8 | 7.9 - | 9.9 | 10.7 | 6.6 - | 11.5 | 7.8 | 5.7 - | 9.1 |  |
|  | %D slope | 10.0 | 8.0 - | 10.6 | 5.6 | 5.0 - | 8.5 | 7.4 | 6.8 - | 8.4 | 5.3 | 4.6 - | 8.5 |  |
|  | SAP DCR | 2.0 | 0.0 - | 7.0 | 0.0 | 0.0 - | 1.0 | 0.0 | 0.0 - | 1.0 | 0.0 | 0.0 - | 1.0 |  |
|  | SAP hurdle | 0.0 | 0.0 - | 1.0 | 0.0 | 0.0 - | 0.0 | 0.0 | 0.0 - | 0.0 | 0.0 | 0.0 - | 0.0 |  |
|  | SAP bridge | 0.0 | 0.0 - | 0.0 | 0.0 | 0.0 - | 0.0 | 0.0 | 0.0 - | 0.0 | 0.0 | 0.0 - | 0.0 |  |
|  | SAP total | 3.0 | 0.0 - | 7.0 | 0.0 | 0.0 - | 1.0 | 0.5 | 0.0 - | 1.0 | 1.0 | 0.0 - | 2.0 |  |
| Risk taking | L CTRCI | 138.3 | 45.2 - | 298.1 | 166.2 | 64.6 - | 273.8 | 138.2 | 50.1 - | 283.8 | 119.3 | 18.3 - | 250.3 |  |
|  | F CTRCI | 4.0 | 4.0 - | 7.0 | 4.0 | 3.0 - | 6.0 | 5.5 | 4.5 - | 6.5 | 5.0 | 2.0 - | 7.0 |  |
|  | D CTRCI | 5.8 | 3.9 - | 12.4 | 4.8 | 2.7 - | 6.1 | 6.2 | 5.0 - | 8.3 | 4.7 | 2.1 - | 12.3 |  |
|  | D/F CTRCI | 1.0 | 0.8 - | 3.1 | 1.0 | 0.9 - | 1.2 | 1.1 | 1.0 - | 1.3 | 1.2 | 1.0 - | 1.8 |  |
|  | %F CTRCI | 5.5 | 4.1 - | 6.5 | 5.0 | 4.3 - | 5.9 | 5.2 | 4.5 - | 7.5 | 5.0 | 3.4 - | 8.5 |  |
|  | %D CTRCI | 0.5 | 0.3 - | 1.0 | 0.4 | 0.2 - | 0.5 | 0.5 | 0.4 - | 0.7 | 0.4 | 0.2 - | 1.0 |  |
|  | L bridge | 262.4 | 108.3 - | 524.5 | 116.8 | 91.5 - | 199.0 | 228.0 | 157.9 - | 432.9 | 282.8 | 151.0 - | 414.2 |  |
|  | F bridge | 5.0 | 5.0 - | 7.0 | 4.0 | 4.0 - | 5.0 | 4.5 | 3.0 - | 5.0 | 3.0 | 2.0 - | 4.0 | P>D**, E>D* |
|  | D bridge | 151.4 | 141.0 - | 200.2 | 115.3 | 77.1 - | 181.6 | 145.1 | 138.6 - | 163.0 | 97.0 | 82.5 - | 123.6 | P>D*, M>D** |
|  | D/F bridge | 30.3 | 20.1 - | 40.0 | 30.1 | 24.9 - | 36.3 | 35.0 | 32.6 - | 43.7 | 31.3 | 29.0 - | 41.2 |  |
|  | %F bridge | 6.5 | 5.1 - | 6.8 | 5.1 | 4.0 - | 6.2 | 4.4 | 3.3 - | 5.2 | 3.6 | 2.4 - | 4.5 |  |
|  | %D bridge | 12.6 | 11.8 - | 16.7 | 9.6 | 6.4 - | 15.1 | 12.1 | 11.6 - | 13.6 | 8.1 | 6.9 - | 10.3 | P>D*, M>D** |
| Shelter seeking | L DCR | 61.4 | 28.2 - | 158.7 | 125.3 | 63.7 - | 199.7 | 83.1 | 35.4 - | 157.2 | 144.0 | 71.8 - | 182.3 |  |
|  | F DCR | 5.0 | 4.0 - | 9.0 | 6.5 | 5.0 - | 10.0 | 9.5 | 6.0 - | 11.0 | 6.0 | 5.0 - | 10.0 |  |
|  | D DCR | 71.5 | 48.6 - | 123.6 | 257.7 | 195.7 - | 272.8 | 237.9 | 189.2 - | 268.1 | 196.5 | 133.9 - | 246.0 |  |
|  | D/F DCR | 13.7 | 9.7 - | 17.9 | 31.1 | 23.2 - | 45.5 | 25.6 | 21.7 - | 29.5 | 24.0 | 22.3 - | 32.8 |  |
|  | %F DCR | 6.8 | 3.7 - | 9.2 | 9.6 | 9.0 - | 10.0 | 9.7 | 7.5 - | 10.1 | 8.5 | 7.4 - | 10.0 |  |
|  | %D DCR | 6.0 | 4.1 - | 10.3 | 21.5 | 16.3 - | 22.7 | 19.8 | 15.8 - | 22.3 | 16.4 | 11.2 - | 20.5 |  |
| Anxiety-like behavior | F risk/shelter index | 0.00 | -0.29 - | 0.27 | -0.30 | -0.43 - | -0.20 | -0.35 | -0.47 - | -0.22 | -0.47 | -0.57 - | -0.33 |  |
|  | D risk/shelter index | 0.33 | 0.10 - | 0.61 | -0.38 | -0.56 - | -0.04 | -0.19 | -0.28 - | -0.05 | -0.32 | -0.55 - | -0.04 |  |
| Impulsive-like behavior | Slope/bridge interval | -0.02 | -0.05 - | -0.02 | -0.05 | -0.08 - | -0.05 | -0.07 | -0.79 - | -0.40 | -0.08 | -0.15 - | -0.06 | P>E*, P>M*, P>D** |
| Other | Grooming | 2.0 | 1.0 - | 2.0 | 2.0 | 1.0 - | 3.0 | 0.5 | 0.0 - | 2.5 | 1.0 | 0.0 - | 1.0 |  |
|  | Urine | 5.0 | 5.0 - | 6.0 | 5.0 | 4.0 - | 5.0 | 2.0 | 1.5 - | 4.5 | 3.0 | 2.0 - | 6.0 |  |
|  | Boli | 0.0 | 0.0 - | 0.0 | 0.0 | 0.0 - | 0.0 | 0.0 | 0.0 - | 0.0 | 0.0 | 0.0 - | 0.0 |  |

Results from the multivariate concentric square field™ (MCSF) test in animals divided by estrus cycle stage: proestrus (P, *n* = 3), estrus (E, *n* = 6), metestrus (M, *n* = 8*)* and diestrus (D, *n* = 11) in Experiment 1. Behavioral parameters recorded during the 20-min trial of the MCSF test. Values represent median with interquartile range. *p<0.05, **<0.01 with *post hoc* Mann-Whitney U-test after p<0.05 with Kruskal-Wallis test. *Abbreviations:* CTRCI, central circle; DCR, dark corner room; D, duration; F, frequency; L, latency; SAP, stretched attend posture; TOTACT, total activity; TOTCORR, total corridor.
