# Supplementary material for: Few long-term consequences after prolonged maternal separation in female Wistar rats
Source: PLoS One. 2017 Dec 21;12(12):e0190042. doi: 10.1371/journal.pone.0190042 (PMC5739456; doi:10.1371/journal.pone.0190042)
Supplement: S3 Table — Body weights (g) of MS15 and MS360 Rcc/Tac animals throughout Experiment 2 (n = 10/group, 40 total). Values represent mean and standard error of the mean (SEM). Six reference time points (HPA, week 1, 6, 9, 14 and 15) were used to compare all subsequent time points. *p<0.05, ***<0.001 post hoc Student’s t-test on whole population after significant main effect of time (p<0.001, repeated measures ANOVA). (DOCX) [file pone.0190042.s006.docx]

**Table S3. Body weights (g) of MS15 and MS360 Rcc/Tac animals throughout Experiment 2.**

|  | **Time point** | **MS15 Rcc** | | **MS360 Rcc** | | **MS15 Tac** | | **MS360 Tac** | |  | **Whole population** | | | |  |  |  |  |  |
| --- | --- | --- | --- | --- | --- | --- | --- | --- | --- | --- | --- | --- | --- | --- | --- | --- | --- | --- | --- |
|  |  | Mean | SEM | Mean | SEM | Mean | SEM | Mean | SEM |  | Mean | SEM | p-values | | |  |  |  |  |
|  | HPA | 232.3 | ± 6.6 | 211.4 | ± 4.5 | 217.6 | ± 2.8 | 219.1 | ± 5.4 |  | 220.1 | ± 2.7 | ref |  | |  |  |  |  |
| Single housing and reversed light/dark cycle acclimatization | | | | | | | |  |  |  |  |  |  |  | |  |  |  |  |
|  | W1 | 242.2 | ± 7.2 | 221.6 | ± 5.2 | 232.5 | ± 2.5 | 231.5 | ± 6.5 |  | 231.9 | ± 2.8 | *** | ref | |  |  |  |  |
|  | W2 | 248.4 | ± 7.8 | 228.3 | ± 5.0 | 236.6 | ± 3.1 | 238.3 | ± 6.4 |  | 237.9 | ± 2.9 | *** | *** | |  |  |  |  |
|  | W3 | 251.3 | ± 7.7 | 235.0 | ± 4.7 | 242.4 | ± 2.9 | 241.3 | ± 6.1 |  | 242.5 | ± 3.0 | *** | *** | |  |  |  |  |
|  | W4 | 259.9 | ± 7.7 | 240.1 | ± 5.9 | 250.9 | ± 2.4 | 250.2 | ± 5.9 |  | 250.3 | ± 2.9 | *** | *** | |  |  |  |  |
|  | W5 | 265.7 | ± 7.9 | 246.5 | ± 6.6 | 257.6 | ± 3.5 | 255.6 | ± 7.2 |  | 256.4 | ± 3.0 | *** | *** | |  |  |  |  |
|  | W6 | 273.9 | ± 9.0 | 253.1 | ± 6.0 | 260.9 | ± 3.3 | 261.0 | ± 6.7 |  | 262.2 | ± 3.3 | *** | *** | | ref |  |  |  |
| Two-week alcohol-deprivation period | | | | |  |  |  |  |  |  |  |  |  |  | |  |  |  |  |
|  | W9 | 289.7 | ± 9.7 | 264.4 | ± 7.5 | 268.2 | ± 3.8 | 271.4 | ± 6.6 |  | 273.4 | ± 3.4 | *** | *** | | *** | ref |  |  |
|  | W10 | 293.3 | ± 10.0 | 268.2 | ± 7.1 | 271.6 | ± 3.8 | 273.2 | ± 6.5 |  | 276.6 | ± 3.8 | *** | *** | | *** | *** |  |  |
|  | W11 | 296.3 | ± 9.5 | 273.0 | ± 6.9 | 276.6 | ± 4.0 | 275.6 | ± 6.4 |  | 280.4 | ± 3.8 | *** | *** | | *** | *** |  |  |
|  | W12 | 299.6 | ± 10.6 | 275.1 | ± 7.2 | 279.3 | ± 4.7 | 279.1 | ± 5.9 |  | 283.3 | ± 3.7 | *** | *** | | *** | *** |  |  |
|  | W13 | 306.9 | ± 10.4 | 278.1 | ± 8.0 | 281.4 | ± 4.9 | 282.9 | ± 7.1 |  | 287.3 | ± 3.9 | *** | *** | | *** | *** |  |  |
|  | W14 | 310.6 | ± 11.7 | 283.9 | ± 7.9 | 282.1 | ± 4.2 | 286.7 | ± 6.9 |  | 290.8 | ± 4.2 | *** | *** | | *** | *** | ref |  |
| Introduction of 5% alcohol to the drinking paradigm | | | | | |  |  |  |  |  |  |  |  |  | |  |  |  |  |
|  | W15 | 309.8 | ± 11.2 | 282.3 | ± 8.2 | 282.2 | ± 4.8 | 282.9 | ± 7.0 |  | 289.3 | ± 4.3 | *** | *** | | *** | *** | * | ref |
|  | W16 | 306.7 | ± 10.7 | 280.1 | ± 7.7 | 280.6 | ± 4.3 | 279.8 | ± 6.4 |  | 286.8 | ± 4.3 | *** | *** | | *** | *** | *** | ** |
|  | W17 | 305.7 | ± 10.0 | 279.2 | ± 7.2 | 278.7 | ± 4.5 | 279.0 | ± 7.6 |  | 285.7 | ± 4.1 | *** | *** | | *** | *** | *** | ** |
|  | W18 | 306.5 | ± 10.4 | 284.4 | ± 7.2 | 279.9 | ± 5.0 | 282.4 | ± 6.9 |  | 288.3 | ± 4.1 | *** | *** | | *** | *** | * |  |
|  | W19 | 310.4 | ± 11.2 | 286.8 | ± 8.2 | 283.7 | ± 5.5 | 285.6 | ± 6.8 |  | 291.6 | ± 4.0 | *** | *** | | *** | *** |  | * |
|  | W20 | 310.6 | ± 11.4 | 288.0 | ± 8.6 | 283.6 | ± 5.2 | 283.2 | ± 6.6 |  | 291.3 | ± 4.3 | *** | *** | | *** | *** |  |  |

Values represent mean with standard error of the mean (SEM). The repeated measures ANOVA showed a main effect of time (p<0.001) with no effect or interaction with rearing condition or supplier. P-values of *post hoc* Student’s paired t-test are shown in the six rightmost columns. Six reference time points (ref) were used for the time-dependent *post hoc* comparisons; each reference point was compared to all subsequent weight measurements. *p<0.05, ***<0.001. HPA, HPA reactivity testing; W, alcohol access week.
